# Characterization of anti-proliferative and anti-oxidant effects of nano-sized vesicles from *Brassica oleracea* L. (Broccoli)

**DOI:** 10.1038/s41598-022-17899-1

**Published:** 2022-08-23

**Authors:** Md Niamat Hossain, Vincenzo De Leo, Rosanna Tamborra, Onofrio Laselva, Chiara Ingrosso, Valeria Daniello, Lucia Catucci, Ilario Losito, Francesco Sollitto, Domenico Loizzi, Massimo Conese, Sante Di Gioia

**Affiliations:** 1 Department of Medical and Surgical Sciences, University of Foggia, Foggia, Italy; 2 Department of Chemistry, University of Bari, Bari, Italy; 3 National Research Council of Italy-Institute for Physical and Chemical Processes (CNR-IPCF S.S. Bari), c/o Department of Chemistry, University of Bari “A. Moro”, Bari, Italy

**Keywords:** Biotechnology, Cancer, Cell biology, Plant sciences

## Abstract

In this in vitro study, we test our hypothesis that Broccoli-derived vesicles (BDVs), combining the anti-oxidant properties of their components and the advantages of their structure, can influence the metabolic activity of different cancer cell lines. BDVs were isolated from homogenized fresh broccoli (*Brassica oleracea* L.) using a sucrose gradient ultracentrifugation method and were characterized in terms of physical properties, such as particle size, morphology, and surface charge by transmission electron microscopy (TEM) and laser doppler electrophoresis (LDE). Glucosinolates content was assessed by RPLC–ESI–MS analysis. Three different human cancer cell lines (colorectal adenocarcinoma Caco-2, lung adenocarcinoma NCI-H441 and neuroblastoma SHSY5Y) were evaluated for metabolic activity by the MTT assay, uptake by fluorescence and confocal microscopy, and anti-oxidant activity by a fluorimetric assay detecting intracellular reactive oxygen species (ROS). Three bands were obtained with average size measured by TEM based size distribution analysis of 52 nm (Band 1), 70 nm (Band 2), and 82 nm (Band 3). Glucobrassicin, glucoraphanin and neoglucobrassicin were found mostly concentrated in Band 1. BDVs affected the metabolic activity of different cancer cell lines in a dose dependent manner compared with untreated cells. Overall, Band 2 and 3 were more toxic than Band 1 irrespective of the cell lines. BDVs were taken up by cells in a dose- and time-dependent manner. Pre-incubation of cells with BDVs resulted in a significant decrease in ROS production in Caco-2 and NCI-H441 stimulated with hydrogen peroxide and SHSY5Y treated with 6-hydroxydopamine, with all three Bands. Our findings open to the possibility to find a novel “green” approach for cancer treatment, focused on using vesicles from broccoli, although a more in-depth characterization of bioactive molecules is warranted.

## Introduction

Plant-derived vesicles (PDVs) can mediate interspecies communication^1^, therefore these natural derivatives are at the forefront of medicine and drug delivery. Nano- and micro-sized PDVs have been isolated from many plant species such as grape^2^, grapefruit^3^, ginger^4^, and broccoli^5^, showing that it is possible to isolate vesicles with a specific proteomic, lipidomic and transcriptomic profile. PDVs offer multiple benefits in terms of size, low toxicity, good tissue specific targeting, excellent environmental safety and a significant potential for large scale production^6^. Considering their wide scale advantages, it can be expected PDVs as next generation therapeutic drug delivery systems for the treatment of a wide range of diseases^7–11^, including cancers^12–15^. For example, PDVs from lemon juice (*Citrus limon* L.) were shown to inhibit cancer cell proliferation in different tumor cell lines both in vitro and in vivo by activating a TRAIL (Tumor Necrosis Factor-Alpha-Related Apoptosis-Inducing Ligand)-mediated apoptotic cell death^14^. Ginger-derived nanoparticles could prevent colitis-associated cancer by reducing pro-inflammatory cytokine levels and mediating intestinal epithelial cells metabolism^16^. PDVs (from grapefruit) could also be loaded with anti-proliferative drugs (methotrexate) and administered to mice with acute colitis thus showing a greater therapeutic index than methotrexate alone^3^. Moreover, grapefruit nanovesicles were used for the intranasal administration of miR-17 leading to the reduction of brain tumor growth in mice^17^.

Cancer cells are characterized by heightened production of reactive oxygen species (ROS), that contribute to tumor growth and proliferation by inducing DNA damage, inflammation, evading immune response, regulating signaling pathways controlling autophagy and apoptosis, angiogenesis, and drug resistance^18^. ROS sources in cancer cells are represented by mitochondrial metabolism, Warburg effect, peroxisome, nicotinamide adenine dinucleotide phosphate hydrogen (NADPH) oxidase and endoplasmic reticulum stress^19^. Nevertheless, cancer cells present also some mechanisms that makes them resistant to oxidative stress through enhanced antioxidant response and increased detoxification capacity, involving both glycolytic and oxidative phosphorylation (OXPHOS) metabolism^20^. This permits cancer cells not to succumb to oxidative stress and to maintain pro-tumorigenic signaling and resistance to apoptosis^19^. Thus, reducing the oxidative stress in cancer is a therapeutic avenue which is currently pursued.

While PDVs from various plants and fruits are able to increase anti-oxidant and anti-inflammatory responses in macrophages and dendritic cells^1,3,5^, mesenchymal stromal cells^21,22^, cardiomyoblast and neuroblastoma cells^23^, the metabolic and anti-oxidant activities of broccoli-derived extracellular vesicles towards cancer cells lines have not been studied yet. Glucosinolates (GSLs) are secondary plant metabolites enriched in Brassicaceae. GSLs, along their products, possess diverse biological activities, including antimicrobial, antioxidant, and anticancer actions^24,25^. Therefore, we isolated and characterized *Brassica oleracea* L*.* (broccoli)-derived vesicles as well as assessed their content in GSLs, and evaluated their uptake by three different cancer cell lines, as well as their anti-proliferative and anti-oxidant effects.

## Results

### Physicochemical characterization of broccoli-derived vesicles (BDVs)

**Figure 1:**
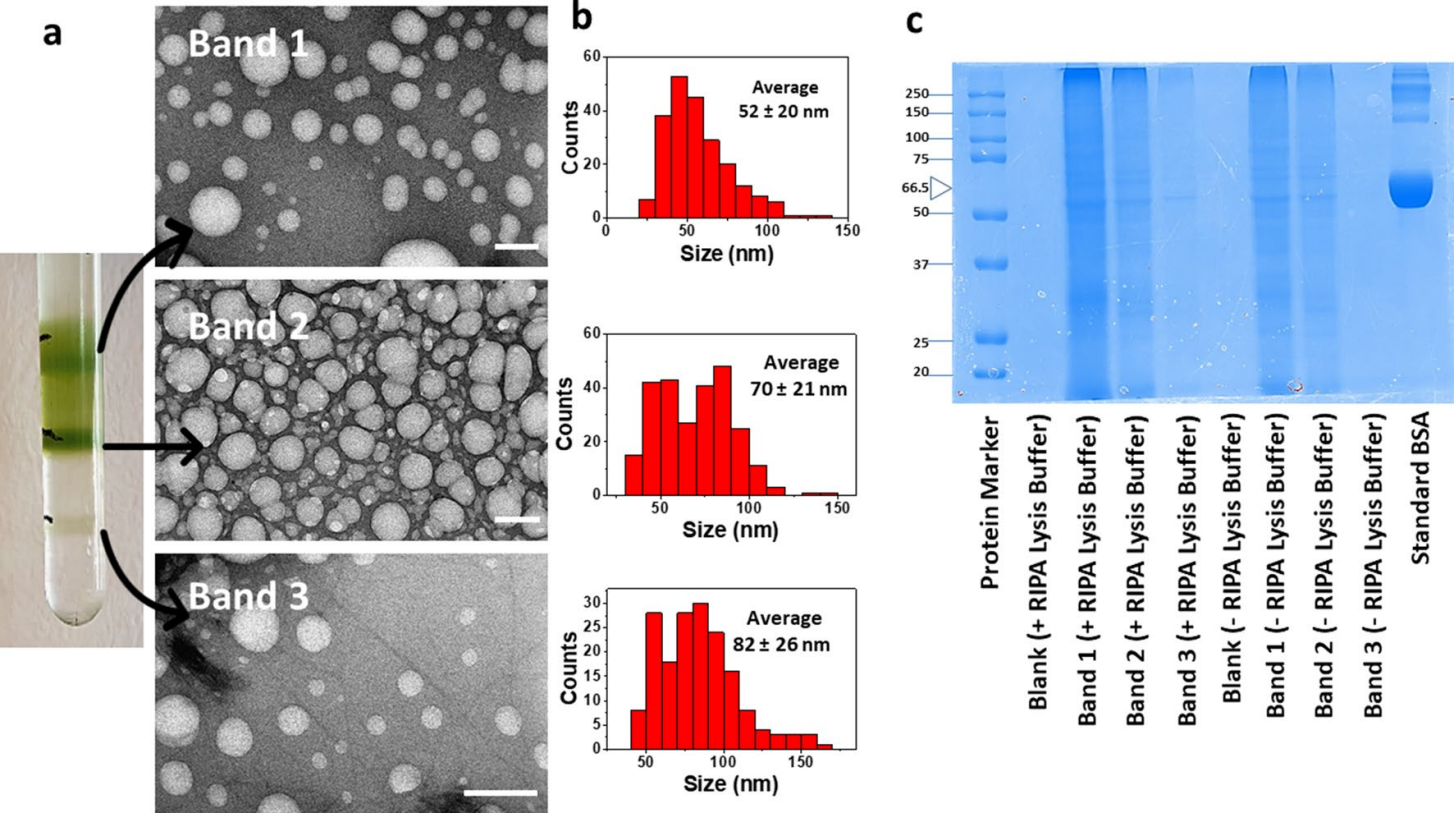
Characterization of BDVs. (**a**) Broccoli-derived vesicles were collected as three bands after discontinuous sucrose gradient ultracentrifugation and analyzed at TEM. Representative TEM images of three BDV bands are shown in the three panels on the right. The scale bars indicate 100 nm. (**b**) TEM-based size distribution measured on 200 BDVs for each band. (**c**) SDS-PAGE of protein extracted from BDVs. Molecular weight of standards is shown on the left as kDa. *BSA* bovine serum albumin, whose MW is indicated as 66.5 kDa by an arrowhead.

*Brassica oleracea* L. vesicles were isolated from the flower heads juice using ultracentrifugation method and purification on a discontinuous sucrose gradient. BDVs mainly accumulated at the 8/30% (Band 1) and 30/45% (Band 2) interfaces of the sucrose gradient while a smaller band was also detected at the 45/60% interface (Band 3) (Fig. 1a). TEM analysis showed the morphology, integrity and size of isolated vesicles (Fig. 1a). Analysis of the size distribution of the nanoparticles showed that BDVs are nanosized and exhibit a fair degree of polydispersity. The BDVs isolated from the various bands are not very dissimilar to each other in size, although an increasing trend can be observed from Band 1 to Band 3 (Fig. 1b). The average size measured by statistical analysis was about 52 nm (Band 1), 70 nm (Band 2), and 82 nm (Band 3), while the Zeta potential measurements established that all BDVs were almost neutral (Table 1).

**Table 1:** Size and zeta potential of isolated BDVs.

Sample	Size (nm)	Zeta potential (mV)
Band 1	52 ± 20	0.00
Band 2	70 ± 21	2.70 ± 0.06
Band 3	82 ± 26	2.8 ± 0.3

Data are shown as mean ± SD (n = 3).

### The three Bands contain different amounts of protein

The protein content, as analysed by the Bradford method, was found to be 1692 ± 182 μg/mL (Band 1), 1201 ± 152 μg/mL (Band 2), and 223 ± 234 μg/mL (Band 3) (n = 2). To see whether proteins were distributed among the outside and inside of vesicles, BDVs were treated with RIPA buffer and compared with untreated vesicles by SDS-PAGE. Figure 1c shows that there were not differences in protein species between RIPA-treated and untreated BDVs. Moreover, the densitometric analysis for each lane determined that RIPA extraction increased only 9%, 3% and 1% the overall intensity of Band 1, Band 2, and Band 3 respectively. Overall, these data indicate that the major proteins were expressed on the vesicle surface of all three Bands.

### Extraction and identification of glucosinolates (GSLs) in BDVs

The occurrence of GSLs in methanol/aqueous extracts obtained from BDVs suspensions was verified by a careful elaboration of the corresponding RPLC-ESI-FTMS and -FTMS/MS (AIF) data. Specifically, ion currents were systematically extracted for a set of exact *m/z* values corresponding to the deprotonated forms of GSLs typical of Brassicaceae vegetables, in accordance with the comprehensive study (involving 25 different species) recently published by Dong et al. and based on the same MS instrumentation used during this work^26^. When chromatographic peaks emerged from the ion current extraction, the occurrence of specific glucosinolate fragments was assessed in the AIF FTMS/MS data acquired in the same retention time intervals of those peaks. If positive, this elaboration provided the final confirmation of the identification of glucosinolates.

As a result, the occurrence of glucobrassicin, glucoraphanin and neoglucobrassicin was confirmed in most samples. The corresponding chromatographic peak areas were then compared with the one related to the internal standard glucocamelinin and an estimate of their concentrations, expressed as glucocamelinin equivalents, was obtained. Considering dilution and pre-concentration steps included in the sample preparation procedure and assuming a quantitative extraction, the concentrations in the original aqueous suspensions of BDVs were calculated and are reported in Table 2.

**Table 2:** Concentrations, expressed as glucocamelinin equivalents and in nanomole/L units, estimated for glucosinolates in the aqueous suspensions corresponding to Bands 1, 2 and 3.

Compound	Band 1	Band 2	Band 3
Glucobrassicin	8.9 ± 1.3	ND	ND
Glucoraphanin	8.7 ± 1.1	0.80 ± 0.08	0.30 ± 0.04
Neoglucobrassicin (N-methoxy-glucobrassicin)	2.6 ± 0.3	ND	ND

Data are shown as mean ± SD (n = 3).

*ND* not detected.

As apparent, all the three GSLs were found in the extract related to Band 1, with concentrations much higher than those observed for the other bands. Actually, as shown in the table, glucobrassicin and neoglucobrassicin could not be detected in extracts related to Bands 2 and 3.

### BDVs reduce the viability of tumor cells from different sites

**Figure 2:**
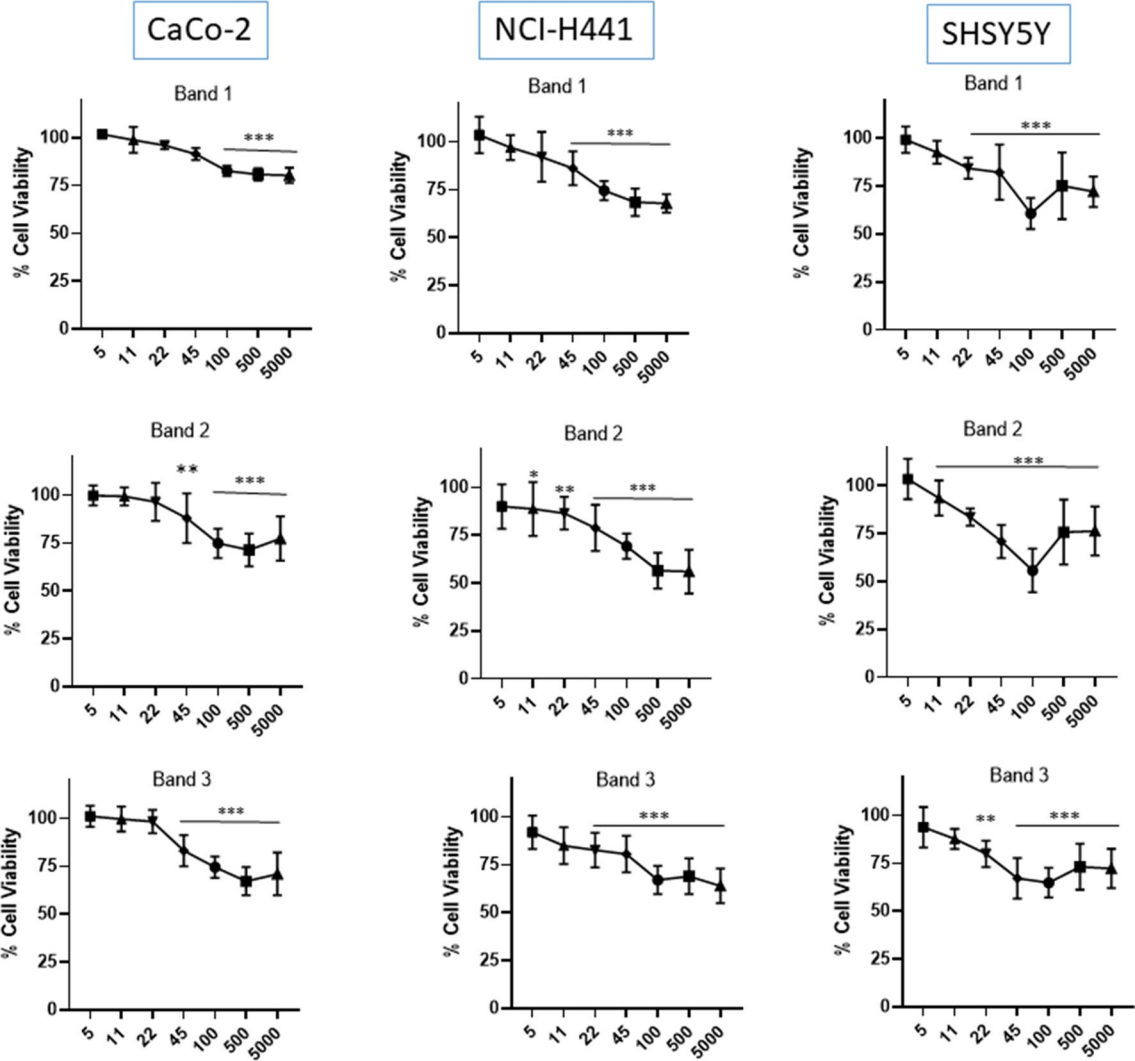
BDVs inhibit the metabolism of tumor cell lines. Cell metabolism was measured by MTT assay after 24 h of treatment with different concentrations of BDVs (indicated as μg/mL). Negative controls are untreated cells (100% of vitality), whereas 10% Triton X-100 was used as positive control and gave an overall vitality of 6.44 ± 1.5% of negative controls (p < 0.001). *p < 0.05; **p < 0.01; ***p < 0.001 vs negative control. Each bar represents the mean ± SD of three independent experiments.

In order to test the ability of *Brassica oleracea* L. vesicles to influence the growth of human tumor cells, NCI-H441 (lung adenocarcinoma), Caco-2 (colorectal adenocarcinoma), and SHSY5Y (neuroblastoma) were treated for 24 h with a concentration range of vesicles (5–5000 μg/mL). The MTT assay showed that BDVs inhibited tumor cell metabolic activity in a dose dependent manner compared with untreated cells (Fig. 2). Overall Bands 2 and 3 were found to be more toxic than Band 1 irrespective of the cell lines.

### Cell uptake of BDVs

**Figure 3:**
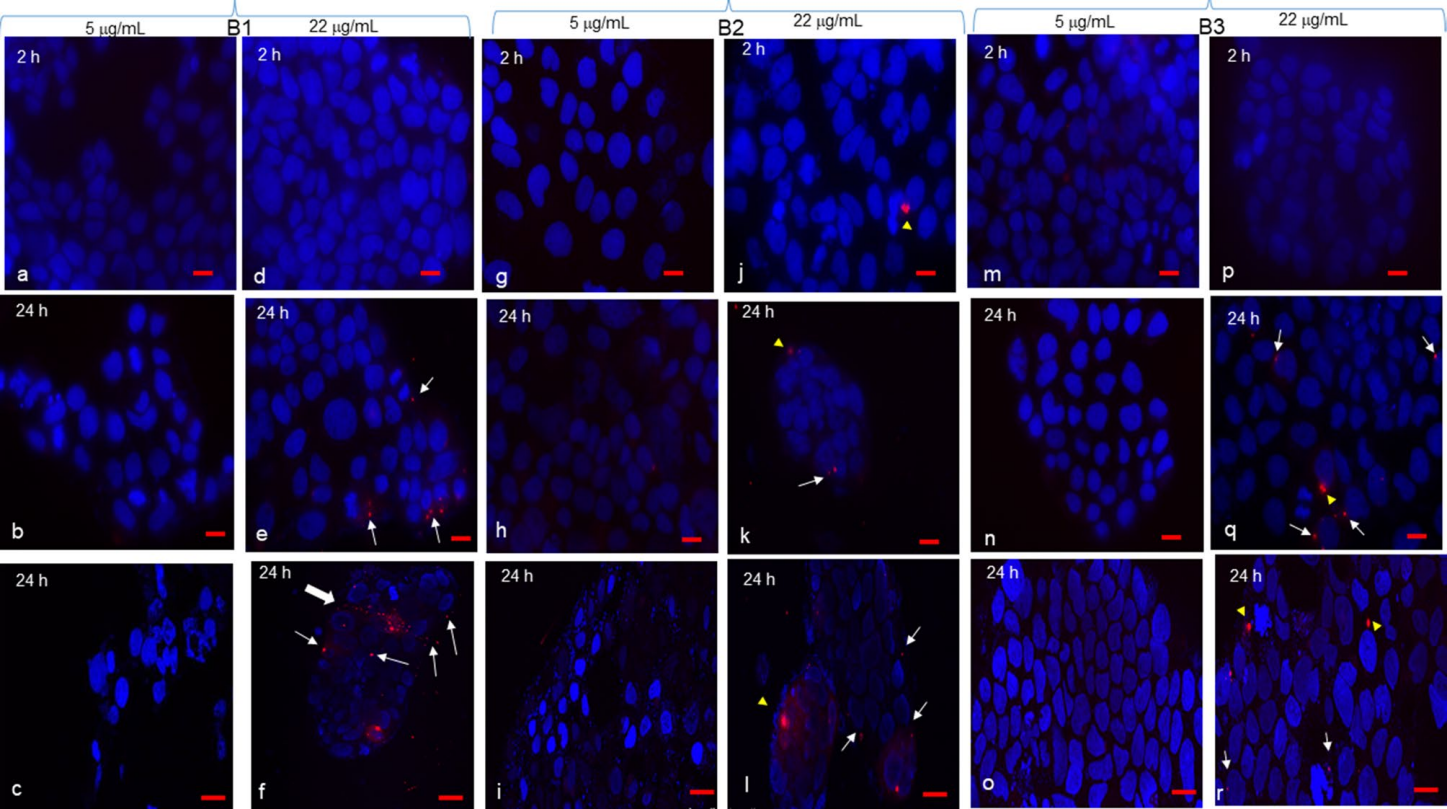
Caco-2 uptake of BDVs. Caco-2 cells were grown on 8-chamber glass tissue culture slides, coverslips mounted, and incubated with Band 1 (**a**–**f**), Band 2 (**g**–**l**), or Band 3 (**m**–**r**) at 5 and 22 μg/mL for 2 or 24 h. After each time point, cells were observed by confocal microscopy (**c**,**f**,**i**,**l**,**o**,**r**) or by epifluorescence (all the remaining panels). White arrows and yellow arrowheads indicate single small and large dots around nuclei respectively. A thick arrow in (**f**) indicates a point of entrance into an island of numerous BDVs. Bar in (**a**,**b**,**d**,**e**,**g**,**h**,**j**,**m**,**n**,**p**,**q**) = 10 μm; bar in (**c**,**f**,**i**,**l**,**o**,**r**) = 25 μm.

In order to see whether these effects were due to the cell uptake of BDVs, we obtained PKH26 fluorescently labelled BDVs, incubated them for 2 and 24 h with Caco-2, NCI-H441 and SHSY5Y cells, and analysed by epifluorescence and confocal microscopy. Two concentrations that were responsible for not more of 20% of cytotoxicity were chosen, i.e. 5 and 22 μg/mL. A fluorescent background signal was not detected when cells were incubated with medium only (not shown). The application of fluorescent BDVs to Caco-2 cells resulted in no or negligible uptake with 5 μg/mL at both time points with all three Band preparations (Fig. 3a–c,g–i,m–o). By using 22 μg/mL, while no uptake was observed at 2 h with all three Bands (Fig. 3d,j,p; just a single cell with B and 2, yellow arrowhead), some cells resulted positive at 24 h as judged by small dots (white arrows) or large dots (yellow arrowheads) around or close to nuclei (Fig. 3e,f,k–l,q–r). Interestingly, while in most cases the punctuate fluorescent signals were located peripherally to the cell islands, we could catch an accumulation of dots in the central part of an island (Fig. 3f).

**Figure 4:**
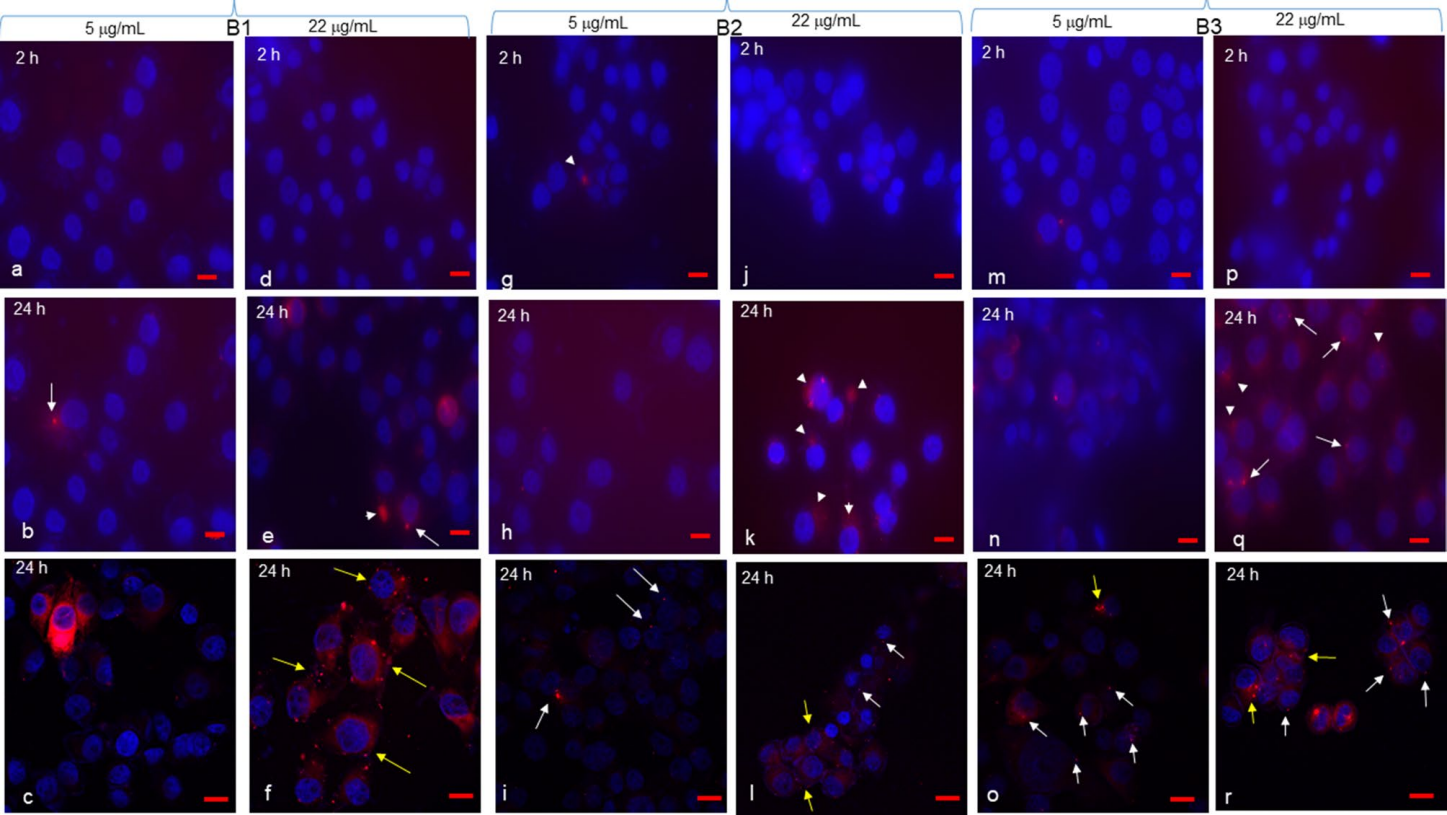
NCI-H441 uptake of BDVs. NCI-H441 cells were grown on 8-chamber glass tissue culture slides, coverslips mounted, and incubated with Band 1 (**a**–**f**), Band 2 (**g**–**l**), or Band 3 (**m**–**r**) at 5 and 22 μg/mL for 2 or 24 h. After each time point, cells were observed by confocal microscopy (**c**,**f**,**i**,**l**,**o**,**r**) or by epifluorescence (all the remaining panels). White arrows indicate single dots around nuclei. Yellow arrows point to a single cell with two or more dots around its nucleus. White arrowheads indicate diffuse perinuclear staining. Bar in (**a**,**b**,**d**,**e**,**g**,**h**,**j**,**m**,**n**,**p**,**q**) = 10 μm; bar in (**c**,**f**,**i**,**l**,**o**,**r**) = 25 μm.

NCI-H441 cells took up BDVs more easily than Caco-2 cells, and at higher extent when challenged for 24 h with vesicles at the concentration of 22 μg/mL (Fig. 4). Interestingly, some cells were associated with two or more dots (yellow arrows) and others with more diffuse perinuclear staining (white arrowheads).

**Figure 5:**
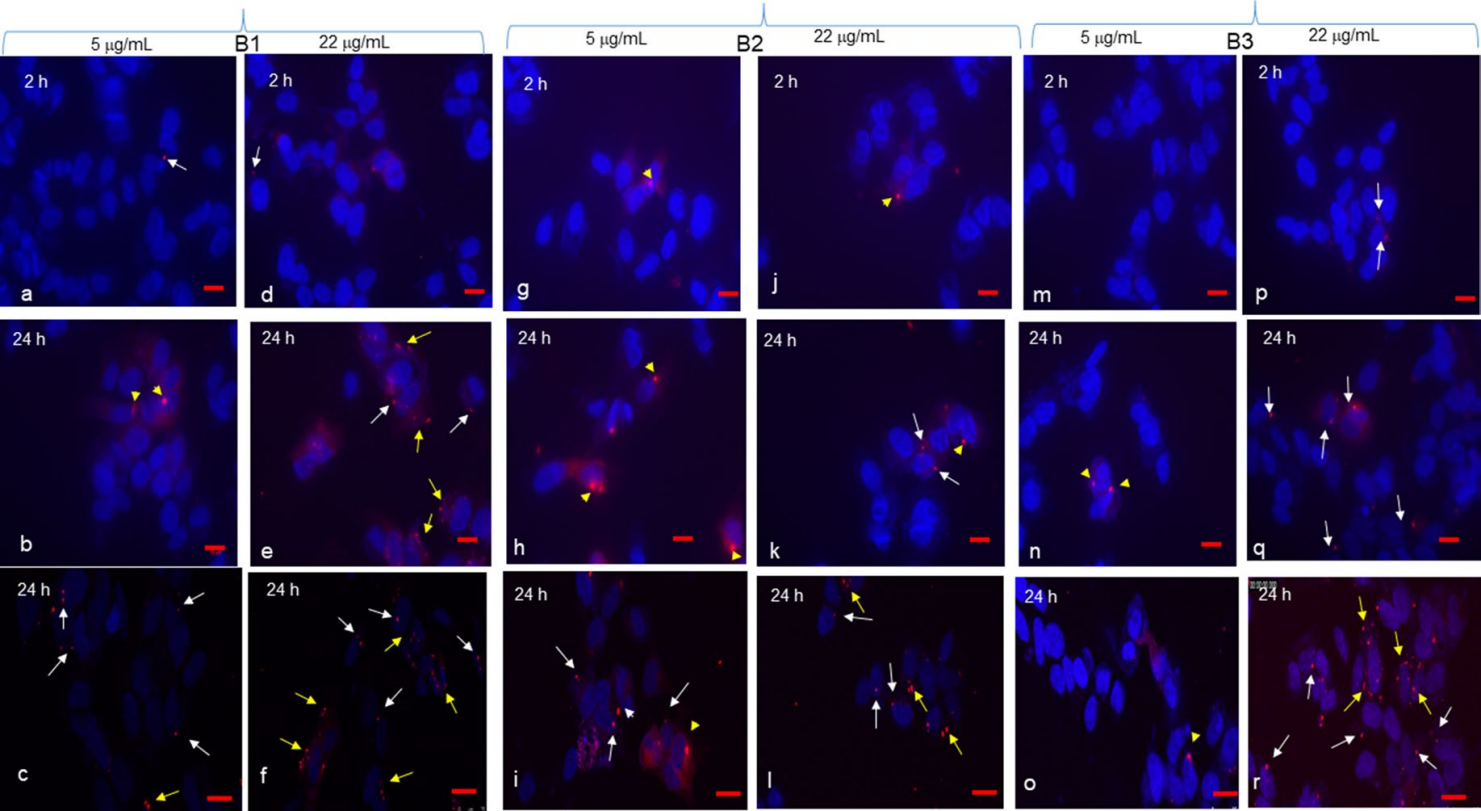
SHSY5Y uptake of BDVs. SHSY5Y cells were grown on 8-chamber glass tissue culture slides, coverslips mounted, and incubated with Band 1 (**a**–**f**), Band 2 (**g**–**l**), or Band 3 (m-r) at 5 and 22 μg/mL for 2 or 24 h. After each time point, cells were observed by confocal microscopy (**c**,**f**,**i**,**l**,**o**,**r**) or by epifluorescence (all the remaining panels). White arrows indicate single dots around nuclei. Yellow arrowheads indicate larger dots. Yellow arrows point to a single cell with two or more dots around its nucleus. Bar in (**a**,**b**,**d**,**e**,**g**,**h**,**j**,**m**,**n**,**p**,**q**) = 10 μm; bar in (**c**,**f**,**i**,**l**,**o**,**r**) = 25 μm.

On the other hand, BDVs were taken up by SHSY5Y cells already with 5 μg/mL (Fig. 5c,i,o). Band 1 and Band 3 at 22 μg/mL were associated with more cells than at 5 μg/mL (Fig. 5, compare f with c, and r with o). Larger dots associated with cells were also observed (yellow arrowheads).

Controls, i.e. cells incubated with medium only, are shown for all cell types in Supplementary Fig. S1.

### Anti-oxidant activity of BDVs

**Figure 6:**
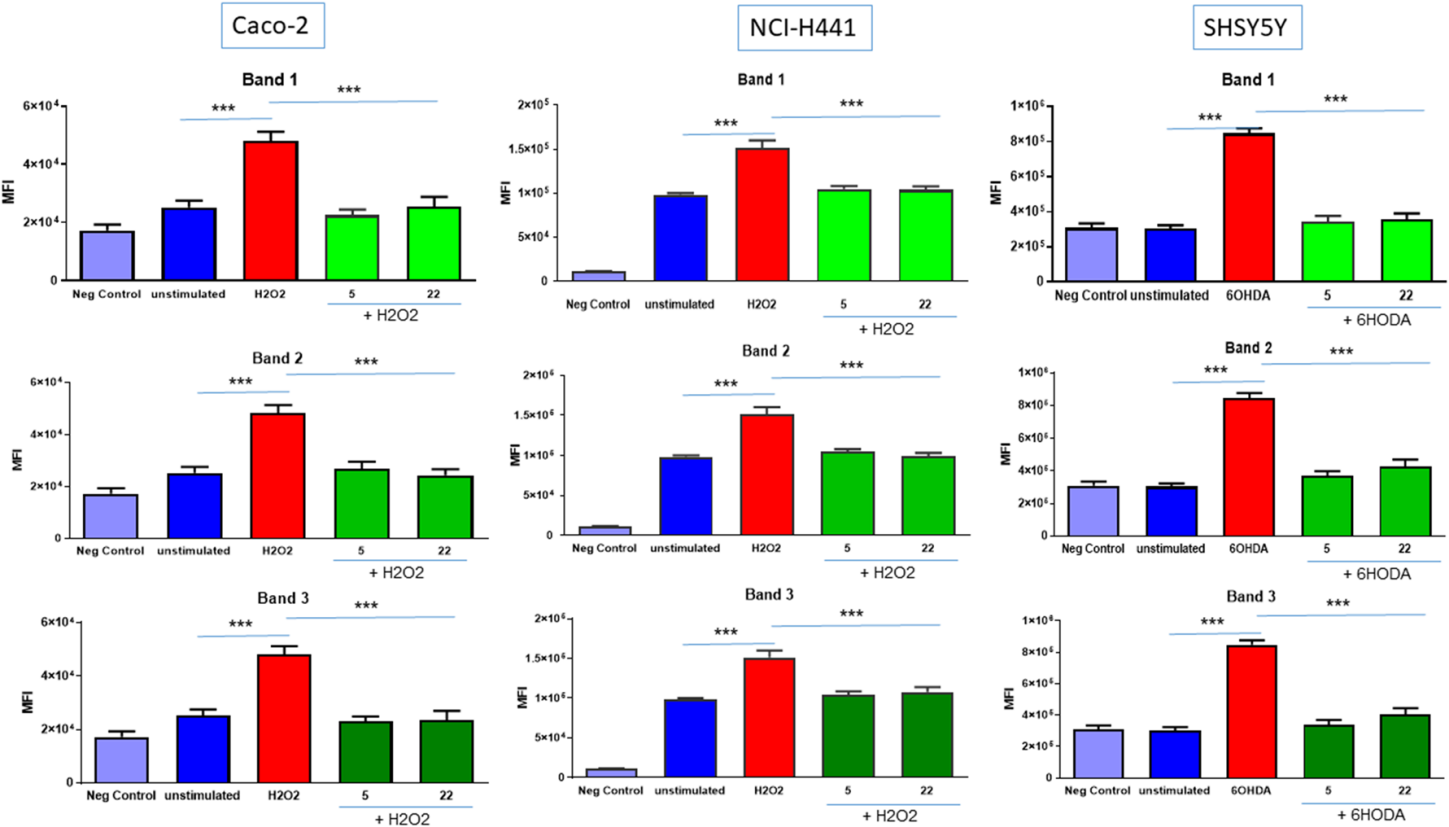
Efficacy of BDVs on oxidative stress. Cells were incubated with 5 or 22 μg/mL BDVs for 24 h before treatment with 100 μM H_2_O_2_ (Caco-2 and NCI-H441) or 100 μM 6-HODA (SHSY5Y) for further 24 h. The mean fluorescence intensity (MFI) was measured by multiwell spectrofluorimeter after incubation with H2DCFDA. Data are shown as mean ± SD of two experiments conducted each in quadruplicate. ***p < 0.001. BDVs at 5 and 22 μg/mL were significantly different as compared with H_2_O_2_- or 6-HODA-stimulated cells, but not towards unstimulated cells.

Finally, we wanted to understand whether BDVs could operate as anti-oxidants in these tumoral cell lines. Caco-2 and NCI-H441 cells are responsive to oxidative stress elicited by H_2_O_2_^27,28^, while SHSY5Y are reactive to the neurotoxin 6-OHDA^29^. Using a ROS-sensitive fluorescent probe (H2DCFDA), we observed that the intracellular levels of ROS were higher in Caco-2 and NCI-H441 cells treated with H_2_O_2_ and SHSY5Y cells treated with 6-OHDA as compared with unstimulated cells (Fig. 6). Pre-treatment with either 5 or 22 μg/mL BDVs determined a reduction of ROS levels when cells were challenged with either H_2_O_2_ or 6-HODA and brought them to those of control unstimulated cells. All Bands were capable to reduce significantly the ROS production.

## Discussion

PDVs are comprised of nano and micro vesicles and have been shown to have powerful anti-inflammatory, anti-oxidant and anti-neoplastic activities^7–9,11,30^. These effects are likely dependent on their cargoes, i.e. lipids, proteins, nucleic acids, and metabolites, although detailed analyses of PDVs biomolecules have been reported only for a few plant species^7^. In vitro and in vivo animal models have therefore shown that PDVs can exert a wealth of therapeutic actions, especially at the level of gastrointestinal tract. Within the number of plants investigated in these studies, broccoli have been used for their production of PDVs only in a single previous work^5^. They isolated BDVs by a sequential centrifugation method followed by column filtration method, obtaining a vesicle population with mean size and Zeta potential of about 32 nm and − 17 mV respectively. Conversely, we obtained three bands from the sucrose gradient ranging from about 50–80 nm in size and with almost zero Zeta potential. Thus, the purification step makes a difference as well as the vegetative stage of the starting matrix, highlighting that a unified protocol would assure to have comparable and reproducible results in the field.

In any case, the isolated BDVs have nanometric dimensions (< 100 nm) which make them very interesting as potential anticancer agents. Nanosized dimensions allows them to penetrate deeply into tumor tissues thanks to the ‘enhanced permeability and retention effect’ (EPR effect), which is dependent on the hyperpermeable tumor vasculature, resulting in an important intratumoral drug accumulation^31^. Moreover, nanoformulations can be incorporated with dyes, contrast agents, drug payloads or inorganic nanoclusters, serving as imaging-guided combinatorial cancer therapeutics^32^.

The MTT assay performed on three different cell lines, belonging to the respiratory tract, colon and central nervous system, shows that BDVs inhibited the metabolic activity of all of them, identifying BDVs as a broad anti-cancer agent. PDVs have been shown to block tumoral cell proliferation by different mechanisms, such as causing cell cycle arrest at G2/M checkpoint^33^, or inciting apoptosis^14^. Whether BDVs block metabolic activity of tumoral cell lines due to the arrest of cell cycle or to apoptosis is something that will be the focus of future studies. However, BDVs were endowed also with anti-oxidant effects and it is known that ROS promote tumor development and progression^19^. Thus, it is likely that BDVs act as anti-tumoral because they are natural anti-oxidants. Sulforaphane (SFN) is a phytocompound belonging to the isothiocyanate family commonly found in many cruciferous vegetables, such as broccoli, brussels sprouts, kale, and cabbages. It has the ability to induce phase II anti-oxidant enzymes and is endowed with anti-inflammatory^34–36^ and anti-proliferative^37–39^ activities. Interestingly it was reported that SFN was enriched in nanoparticles compared to that in microparticles isolated from broccoli-derived lipid extracts^5^. In accordance with this evidence we found that all three nanostructured bands were endowed with anti-oxidant and anti-proliferative activities. We did not detect SFN in our BDVs, although we appreciated the presence of its precursor glucoraphanin, particularly in Band 1. Band 1 also contains appreciable quantities of glucobrassicin and neoglucobrassicin, which were not detected in Band 2 and Band 3. The fact that all three bands had relevant biological activities, make other biological constituents relevant in this context.

It must be said that as to the analytical determination of anti-oxidant molecules, this study is the first in which BDVs are used as source for the determination of GSLs and SFN. So far, the huge body of scientific literature concerning the determination of bioactive compounds from broccoli was produced using crude tissues. For instance, levels of GSLs can fluctuate dramatically even between cultivars and different growing conditions^40,41^. Also, the stability of these bioactive compounds can be influenced both by different processes used during their extraction and by their storage. Recently, it has been demonstrated how the cutting styles affect the stability and accumulation of bioactive compounds in broccoli^42^. Probably, in our experimental procedures, the mechanical procedure of extraction has caused a massive reduction of glucobrassicin and neoglucobrassicin in Band 2 and Band 3. Moreover, the effect of storage should be taken in account since in an another study it has been observed that the freezing process can decrease both glucoraphanin and SFN levels^43^. This decrease is likely due to the inhibition of hydrolysis of glucoraphanin by myrosinase. Taken in account these results, we hypothesized that in our study, SFN was not detectable as a consequence of freezing process.

Except the protein content, we could not determine lipid and RNA molecules, thus we have to further investigate BDV payload to fully correlate with biological activities. As to the proteins expressed by PDVs, interestingly, it has been observed that garlic-derived vesicles are uptaken by HepG2 hepatocellular carcinoma cells through the interaction of cellular CD98 and lectin-type proteins located on the vesicle surface^44^. The exact protein profile of BDVs will unveil how they interact with cancer cells and allow to better comprehend their full therapeutic potential.

Anti-metabolic and anti-oxidant activities of BDVs are mediated by their cell uptake. The three cell lines behaved differently in this context as concerning dose and time. Caco-2 cells were the least amenable to BDV internalization which was evident just with 22 μg/mL at 24 h, likely due to island-like growth of this cell line. However, there was indication that higher uptake with nano-sized Band 1 was observed when intercellular junctions are altered. Some nanoparticles have been shown to induce a reversible loosening of tight junctions by unknown mechanisms^45,46^. While SHSY5Y took up BDVs already with 5 μg/mL, an intermediate behavior was shown by NCI-H441 cells. The uptake of nano- and micro-sized vesicles differ, ranging from phagocytosis of ~ 188 nm ginger vesicles by colon cancer cells^16^ to garlic EVs of < 150 nm that are internalized by macropinocytosis in hepatocytes^44^. Thus, our nano-sized BDVs were taken up likely by an array of mechanisms, including clathrin-mediated endocytosis, phagocytosis, macropinocytosis, and plasma or endosomal membrane fusion of vesicles^47^.

## Conclusions

PDVs that carry chemicals, proteins, and nucleic acids from vegetables and fruits may provide far more treatment options than synthetic drugs and protein medicine^8^. Plants represent green, sustainable and renewable sources of nanovesicles, and this can ensure a constant and never-ending production, in turn providing bioproducts that are immediately available at low dosages, stable and more effective than the current products, and therefore more suitable for clinical use and the market^48^. Here we show that it is possible to isolate nano-sized BDVs from *Brassica oleracea* L.. These vesicles were endowed with anti-proliferative and anti-oxidant activities on different cancer cells, dependent on cell uptake. However, the clinical translation of BDVs into therapy application is still a long way to go. As a kind of biological medicine, production standard, shelf-life, and quality control remain to be determined. In vivo pre-clinical animal models of cancer need to be investigated. BDV nanovesicles are apt to be further exploited for cancer theranostics, although bioactives should be identified yet.

## Methods

### Chemicals

LC–MS grade acetonitrile (Cat# 1.00029), water (Cat# 270733), and methanol (Cat# 34860), used for Reversed Phase Liquid Chromatography- Electrospray Ionization-Mass Spectrometry (RPLC-ESI–MS) separations and/or for glucosinolate extraction, and LC–MS grade formic acid, used as mobile phase additive, were purchased from Merck (Milan, Italy). Standard glucocamelinin (Cat# PHL85744), used as internal standard for glucosinolate quantification, was purchased from Merck (Milan, Italy).

### Isolation and purification of *Brassica oleracea* L. (Broccoli)-derived vesicles (BDVs)

BDVs were isolated from the flower heads juice. All methods were carried out in accordance with relevant institutional, national, and international guidelines and legislation guidelines complying with with the Convention on Biological Diversity (https://www.cbd.int/convention/) and the Convention on the Trade in Endangered Species of Wild Fauna and Flora (https://cites.org/eng). Broccoli (580.0 g), obtained from a local grocery store, was carefully washed three times with distilled water to remove dust, soil, and pesticides and then finally washed with 1 × phosphate buffered saline (PBS), produced from DPBS 10 × (Corning, Cat# 20-031-CV). After the final washing, the broccoli was blended under maximum power for 10 min (2 min on, 2 min pause) in a juicer mixer (Ariete Juicer Mixer; Centrika Slow Juicer metal 177/1, Cat# 00C017710AR) to obtain juice. The collected juice (about 280–300 mL) was always maintained at 4 °C (using an ice bucket) and passed through a sterile cotton-gauze which is clamped to the lower edge of a bucket, so as to exclude rough residues. Then the juice was sequentially centrifuged (Thermo Scientific Biofuge Stratos Benchtop Centrifuge, rotor# 3334) at 1000×*g* for 10 min, 3000×*g* for 20 min and 10,000×*g* for 40 min at 4 °C to remove cell debris, fibers, various aggregates and intracellular organelles. The supernatant was then ultracentrifuged (Beckman Optima L-90 K) at 146,000×*g* for 60 min at 4 °C using a Type SW 32 Ti Beckman rotor and the pellet was suspended in 1 × PBS.

For purification of BDVs, the suspension was transferred to a discontinuous sucrose gradient (IBI Scientific, Cat# IB37160) (8%, 30%, 45% and 60% [g/v]) and ultracentrifuged at 146,000×*g* for an additional 60 min at 4 °C using a Type SW 32 Ti Beckman rotor. The bands between 8/30%, 30/45%, and 45/60% layers, which correspond to Band 1, Band 2 and Band 3, respectively, were recovered, ultracentrifuged for 60 min in PBS 1 × and pellets were collected and resuspended in 10% mannitol (Sigma-Aldrich, Cat# 15719) in 1 × PBS. The suspensions were then stored at − 80 °C until use.

### Physicochemical characterization of BDVs

BDVs particle Zeta potential was measured by laser doppler electrophoresis (LDE) using a Zeta sizer Nano ZS and in diffusion barrier mode with water^49^.

The TEM analysis was done by a method previously described with modification^50^. In brief, samples were prepared by dropping the BDV aqueous suspension (2 μL) on a 400-mesh amorphous carbon-coated Cu grid (Agar Scientific Ltd, Stansted, UK, Cat# S160-4) and letting the solvent evaporate. Sample on the grid was left to dry overnight and finally stored in a vacuum chamber until analysis. The samples were stable under the electron beam and did not degrade within the typical observation times. No staining was used in these experiments. Micrographs were recorded using a Jeol JEM-1011 microscope working at an accelerating voltage of 100 kV and acquired by an Olympus Quemesa Camera (11 Mpx). Size statistical analysis (BDV average size and size distribution) of each sample (Band 1, Band 2 and Band 3) was performed on 200 nanostructures by means of a freeware Image J analysis program (National Institutes of Health, USA).

### Determination of protein concentration and SDS–PAGE

The protein concentration of the samples was determined using a Coomassie Plus (Bradford) assay kit (Thermo Scientific, Cat# 23238). An aliquot (25 μl) of each band sample was lysed with 10 μl of Radioimmunoprecipitation assay (RIPA) lysis buffer (EMD Millipore Corporation, Cat# 20–188). After centrifugation at 12,000 rpm for 10 min at 4 °C, the supernatant was considered for the determination of protein concentration as per manufacturer's instruction. Proteins were electrophoresed on 10% SDS–polyacrylamide gels and stained with Coomassie blue. Bovine serum albumin was used as standard. Bio-Rad Precision plus protein standards (Cat# 161-0374) was used as marker. Integrated intensity for each lane was evaluated by the ImageJ software. Uncropped gel is provided in Supplementary Fig. S2.

### Extraction of GSLs and sample preparation

Aliquots of the PBS suspensions obtained from pellets recovered from Bands 1, 2 and 3 were mixed with methanol to obtain a final methanol/water 70:30 (v/v) solvent composition, corresponding to the one previously employed by Cataldi et al.^51^ for the extraction of GSLs from vegetal matrices. The resulting suspensions were sonicated for 20 min at 40 °C and then centrifuged at 12,000 rpm; afterwards, the supernatant was recovered and stored at + 4 °C for 24 h. Since mannitol crystals, previously added as a cryopreservative, precipitated upon prolonged low temperature storage, a new centrifugation at 12,000 rpm was carried out. The resulting supernatant was first pre-concentrated by a factor 2 through partial solvent evaporation, then it was stored at − 20 °C until RPLC-ESI–MS analysis was performed.

Just before the analysis, the methanol/water extracts referred to Bands 1, 2 and 3 were fortified with glucocamelinin (final concentration 5.76 μmol/L), that was adopted as internal standard to estimate the concentration of glucosinolates extracted from BDVs, under the assumption that electrospray ionization yields for different glucosinolates (and, consequently, the MS response vs concentration dependence) are comparable.

### RPLC–ESI–MS instrumentation and operating conditions

RPLC–ESI(-)–MS analyses were performed using an LC–MS platform including an Ultimate 3000 HPLC quaternary chromatographic system interfaced to a Q-Exactive high resolution quadrupole-Orbitrap mass spectrometer (Thermo Fisher, West Palm Beach, CA, USA), that was used for Fourier-transform MS (FTMS) and for Higher-energy Collisionally-induced Dissociation tandem MS acquisitions (HCD-FTMS/MS).

RPLC separations of BDV extracts were performed using a C18 Ascentis Express column (15 cm length, 2.1 mm internal diameter) packed with core–shell 2.6 μm particles (Supelco, Bellefonte, PA, USA) and operated at a 0.2 mL/min flow; 5 μL sample volumes were injected. The following multi-step binary elution gradient, based on an water as phase A and acetonitrile as phase B, both containing 0.1% (v/v) formic acid, was adopted for lipid separation: 0–2 min) isocratic at 1% B; 2–3 min) linear increase of B from 1 to 2%; 3–5 min) isocratic at 2% B; 5–10 min) linear increase of B from 2 to 10%; 10–20 min) linear increase of B from 10 to 30%; 20–30 min) linear increase of B from 30 to 80%; 30–40 min) isocratic at 80% B; 40–50 min) linear decrease of B from 80 to 1%; 50–60 min) reconditioning at 1% B.

The Heated ElectroSpray Ionization (HESI) interface (Thermo Fisher, West Palm Beach, CA, USA) mounted on the Q-Exactive spectrometer was adopted to ionize analytes eluted from the chromatographic column and transfer the resulting ions into the mass spectrometer.

The parameters of the HESI interface and of the ion optics of the Q-Exactive spectrometer were set as follows: sheath gas (nitrogen) flow rate) 40 a.u.; auxiliary gas (nitrogen) flow rate) 15 a.u.; spray voltage) − 3 kV; capillary temperature) 320 °C; S-lens RF level 60. The spectrometer was operated in negative polarity at its maximum resolving power (140,000 at m/z 200) and high-resolution spectra were acquired in a 300–700 m/z interval. HCD–FTMS/MS acquisitions were performed systematically on all ions generated into the HESI source by using the All-Ion Fragmentation (AIF) acquisition mode, enabling the retrieval of fragmentation (HCD–MS/MS) spectra of eluted compounds with an untargeted approach. AIF scans were acquired at a 70,000 resolving power in a 50–700 m/z interval, using a normalized collisional energy (NCE) of 35 units. During FTMS measurements the Orbitrap fill time was set to 100 ms and the Automatic Gain Control (AGC) level was set as 1 × 10^6^. Before analyses the spectrometer was calibrated by infusing, at a 5 μL/min flow rate, calibration solutions provided by the instrument manufacturer for positive or negative polarity acquisitions. As a result, a mass accuracy always better than 5 ppm was achieved.

### Cell cultures

Caco2 cells (ATCC, Manassas, VA, USA) were grown in DMEM with 4.5 g/L glucose, l-glutamate and sodium pyruvate supplemented with 10% Fetal Bovine Serum (FBS, Corning, Cat# 35-010-CV), 1% penicillin and streptomycin, 25 µg/mL amphotericin B, and 2 mM L-Glutamine. NCI-H441 cells (ATCC) were grown in RPMI-1640 (Corning, Cat# 10-040-CV) with l-glutamine, supplemented with 10% FBS, 1% penicillin and streptomycin (EuroClone, Cat# ECB3001D), 25 µg/mL amphotericin B (Corning, Cat# 30-003-CF), and 2 mM l-glutamine (EuroClone, Cat# ECB3000D). SHSY5Y cells (kindly provided by Dr. Maria Lasalvia, University of Foggia) were grown in Dulbecco's Modified Eagle's Medium Nutrient Mixture F-12 Ham (Sigma-Aldrich, Cat# D8062), 20% FBS, 1% non-essential amino acids, 2 mM l-glutamine, 1% penicillin and streptomycin, and 25 µg/mL amphotericin B. The cells were incubated at 37 °C in a humidified incubator with 5% CO_2_ and the culture medium was refreshed every other day.

### Viability assay (MTT assay)

Cell viability was assessed with Methyl-thiazol-tetrazolium (MTT, Sigma-Aldrich, Cat# MKBL6157V) assay as detailed previously^14^. For the assessment, Caco-2, NCI-H441, or SHSY5Y cells were seeded in quadruplicate at a density of 2 × 10^4^ or 1 × 10^4^ in a 96-well plate respectively and exposed to escalating doses of BDVs (from 5 to 5000 μg/mL) for 24 h. After which the BDVs-containing medium was removed and cells were thoroughly rinsed once with 1 × PBS. Cells were then incubated with 100 μL of MTT solution (0.5 mg/mL) at 37 °C for 4 h until a purple precipitate was visible. Thereafter, the media were discarded and 100 μl dimethyl sulfoxide (DMSO, Corning, Cat# 25-950-CQC) was added to each well prior to spectrophotometric measurements at 595 nm. Means and standard deviations were generated from three independent experiments and reported as the percentage of viability versus control (untreated cells). Triton X-100 (10% v/v solution, BioUltra, Cat# 93443) treated cells were used as positive control. Untreated cells were used as a negative control.

### BDVs labelling and cell uptake

To verify that BDVs can be taken up by human cells, BDVs were stained with PKH26 red dye (Sigma-Aldrich, Cat# MIDI26) followed by incubation with cells. BDVs were isolated as described above and labeled with PKH26 for 5 min at room temperature as previously described with minor modifications^52^. Labelling was stopped by adding 10% Bovine Serum Albumin (BSA, BioReagent, Cat# a8806). All unlabeled dye was washed away by ultracentrifugation at 100,000×*g* for 70 min, and labelled BDVs pellets were re-suspended in 1 × PBS.

NCI-H441, Caco-2 and SHSY5Y cells were seeded in 8-chamber glass tissue culture slides (Sarstedt, Nümbrecht, Germany, Cat# 94.6170.802) and incubated overnight in growth medium. Then the cells were treated with 5 μg/mL and 22 μg/mL of PKH26-labelled BDVs for 2 or 24 h at 37 °C. Negative controls for background fluorescence were cells incubated with medium only for 2 or 24 h**.** After co-culture, uptake was stopped by washing and the cells were fixed with fix solution in 1 × PBS for 5 min at room temperature. After washing twice with 1 × PBS, finally the cells were coverslip-mounted with mounting medium containing 4-,6-diamidino-2-phenylindole (DAPI, H-1200; Vector Laboratories, Burlingame, CA, USA, Cat# H-1200–10) for nuclear staining and analysed by fluorescence microscopy. Images were acquired on the Nikon Eclipse Ni Fluorescence Microscopy system. Acquisition, storage and analysis of data were performed with NIS Elements imaging software (Nikon Europe B.V.).

For confocal microscopy, images were acquired on the Leica TCS SP8 confocal laser scanning microscopy system. Acquisition, storage and analysis of data were performed with LasX software (Leica Microsystems GmbH, Wetzlar, Germany).

### Determination of anti-oxidant activity

To evaluate the efficiency of anti-oxidant activity, Caco-2, NCI-H441 and SHSY5Y cells were pretreated with BDVs (5 and 22 μg/mL) for 24 h. Then, in order to induce oxidative stress, Caco-2 and NCI-H441 cells were treated with 100 µM hydrogen peroxide (H_2_O_2_, Sigma-Aldrich, Cat# H1009) for 24 h^27,28^, while SHSY5Y cells were challenged with 100 µM 6-hydroxydopamine (6-OHDA, Sigma-Aldrich, Cat# H4381) for 24 h^29^. Reactive oxygen species (ROS) production was evaluated by fluorimetry by using the 2′,7′-Dichlorodihydrofluorescein diacetate (H2DCFDA, Sigma-Aldrich, Cat# D6883)), as previously described^28^. The dose and timing of H_2_O_2_ and 6-OHDA incubation with cells were chosen based on preliminary experiments that evaluated significant ROS production in the absence of overt reduced viability. Briefly, Caco-2, NCI-H441 and SHSY5Y cells were washed and then incubated with 10 µM H2DCFDA for 90 min at 37 °C and the fluorescent signal of DCF obtained from the conversion of H2DCFDA by intracellular ROS produced was measured at excitation/emission wavelengths of 485/530 nm using a Fluorescent Plate Reader (FilterMax F5 Multi-Mode Microplate Reader (Molecular Devices, Beckman Coulter, CA, USA). The results were expressed as mean fluorescence intensity (MFI). The assay was performed two times in quadruplicates.

### Statistical analysis

Data were analyzed by one-way analysis of variance (ANOVA) using Prism for Windows, version 8.0.1, GraphPad Software Inc., San Diego, CA, USA. Tukey’s Multiple Comparison Test was used to examine differences between group means. A P value < 0.05 was considered statistically significant. Data are shown as means ± SD.

## Supplementary Information

Supplementary Figure 1.

Supplementary Figure 2.

## Data Availability

All data generated or analyzed during this study are included in this published article.
