# Supplementary material for: Characterization of anti-proliferative and anti-oxidant effects of nano-sized vesicles from Brassica oleracea L. (Broccoli)
Source: Sci Rep. 2022 Aug 23;12:14362. doi: 10.1038/s41598-022-17899-1 (PMC9399156; doi:10.1038/s41598-022-17899-1)
Supplement: Supplementary file 1 — Supplementary Figure 1. [file 41598_2022_17899_MOESM1_ESM.pdf]

Characterization of anti-proliferative and anti-oxidant effects of nano-sized vesicles from *Brassica oleracea* L. (Broccoli)

Md Niamat Hossain[1], Vincenzo De Leo[2], Rosanna Tamborra[1], Onofrio Laselva[1], Chiara Ingrosso[3], Valeria Daniello[1], Lucia Catucci[2], Ilario Losito[2], Francesco Sollitto[1], Domenico Loizzi[1*], Massimo Conese[1*] and Sante Di Gioia[1]

[1]Department of Medical and Surgical Sciences, University of Foggia, Foggia, Italy.

[2]Department of Chemistry, University of Bari, Bari, Italy.

[3]National Research Council of Italy-Institute for Physical and Chemical Processes (CNR-IPCF S.S. Bari), c/o Department of Chemistry, University of Bari "A. Moro", Bari, Italy

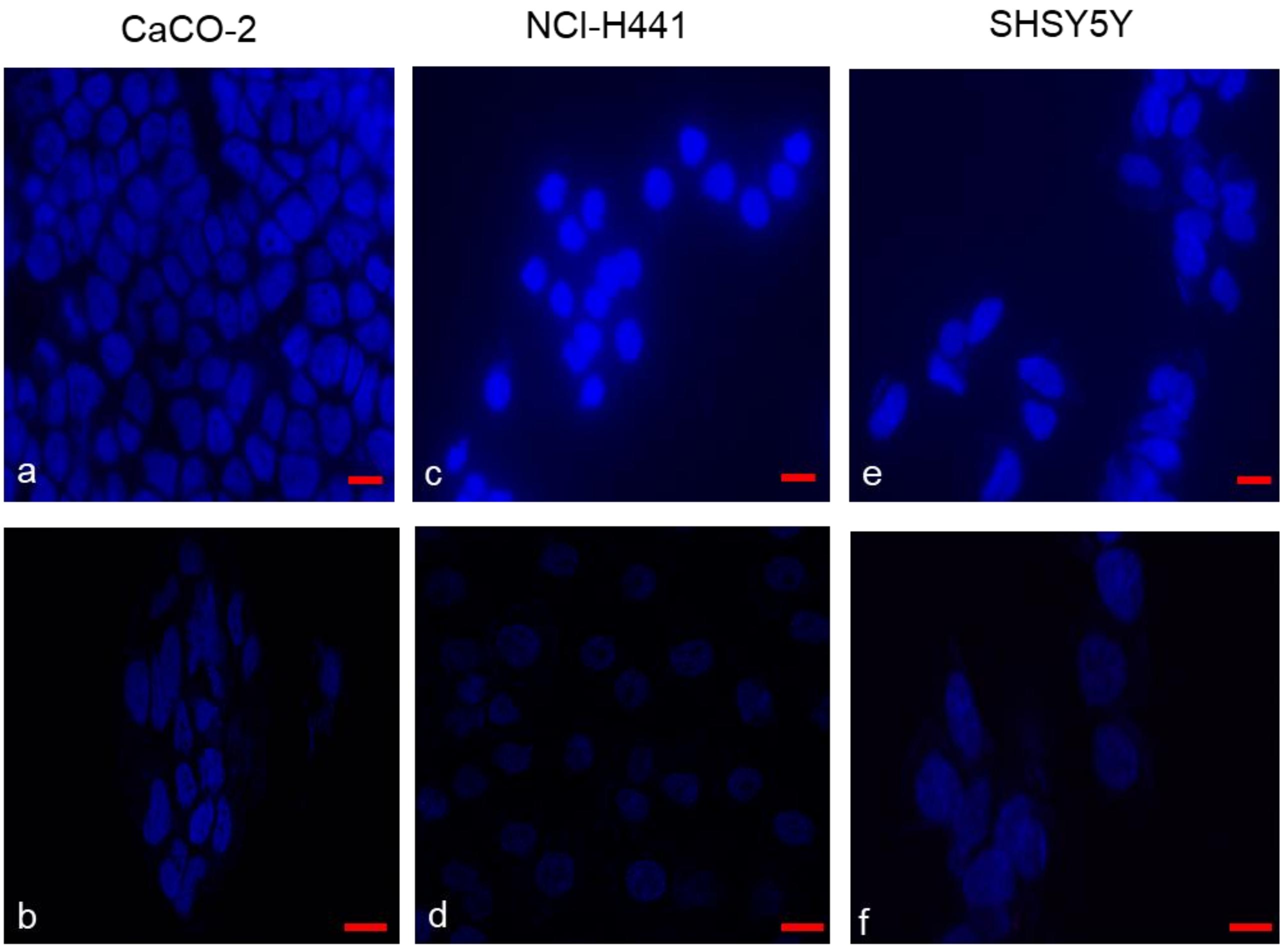

S1 Figure. Negative controls of cell types shown in Figures 3, 4, and 5. Cells were incubated with medium only and stained with DAPI. a, c, and e represents the negative control from epifluorescence microscopic observation. Bar = 10 μm. b, d, and f represent the negative control from confocal microscopic observation. Bar= 25 μm.
