# Supplementary material for: Characterization of anti-proliferative and anti-oxidant effects of nano-sized vesicles from Brassica oleracea L. (Broccoli)
Source: Sci Rep. 2022 Aug 23;12:14362. doi: 10.1038/s41598-022-17899-1 (PMC9399156; doi:10.1038/s41598-022-17899-1)
Supplement: Supplementary file 2 — Supplementary Figure 2. [file 41598_2022_17899_MOESM2_ESM.pdf]

Characterization of anti-proliferative and anti-oxidant effects of nano-sized vesicles from *Brassica oleracea* L. (Broccoli)

Md Niamat Hossain[1], Vincenzo De Leo[2], Rosanna Tamborra[1], Onofrio Laselva[1], Chiara Ingrosso[3], Valeria Daniello[1], Lucia Catucci[2], Ilario Losito[2], Francesco Sollitto[1], Domenico Loizzi[1*], Massimo Conese[1*] and Sante Di Gioia[1]

[1]Department of Medical and Surgical Sciences, University of Foggia, Foggia, Italy.

[2]Department of Chemistry, University of Bari, Bari, Italy.

[3]National Research Council of Italy-Institute for Physical and Chemical Processes (CNR-IPCF S.S. Bari), c/o Department of Chemistry, University of Bari "A. Moro", Bari, Italy

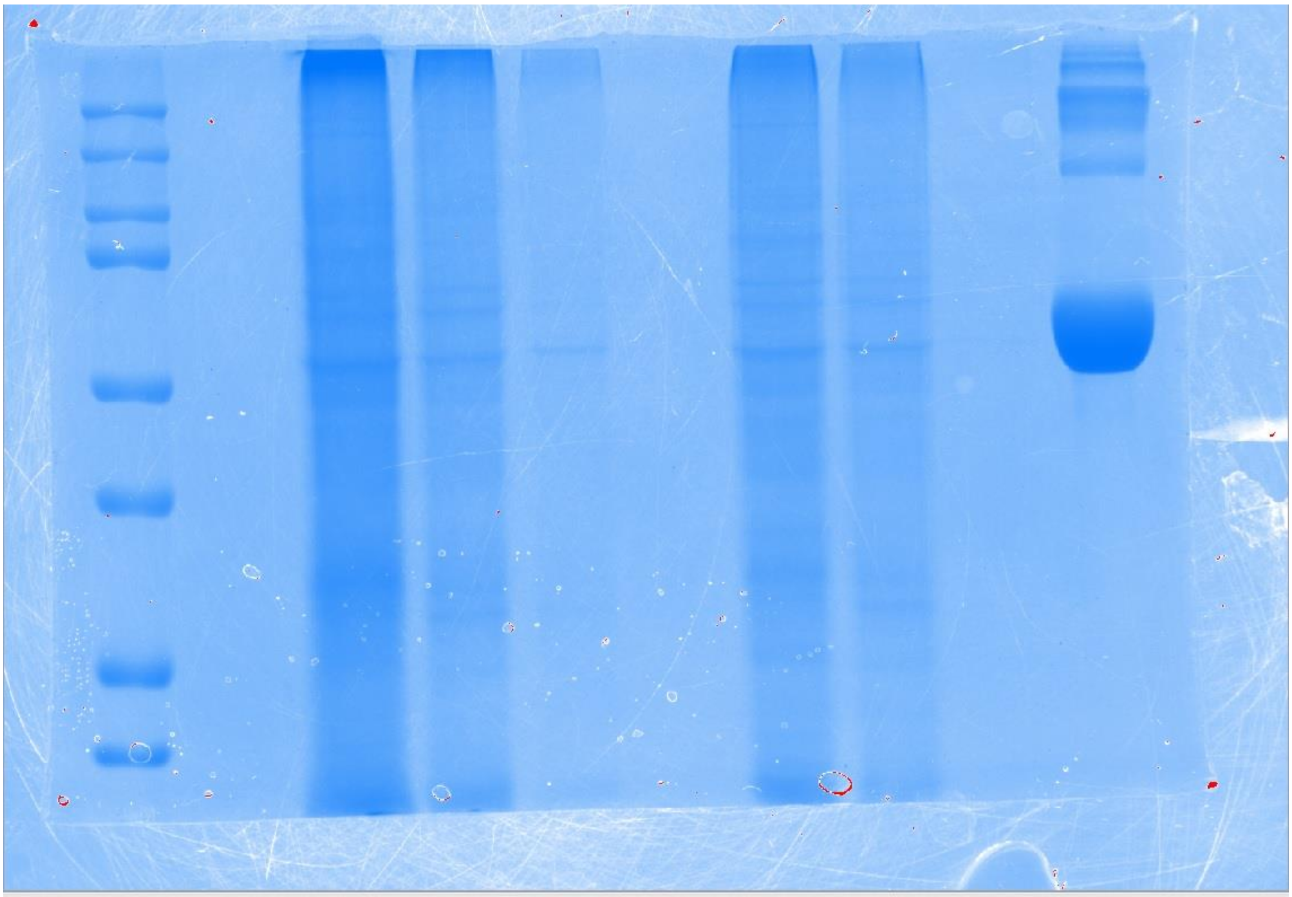

S2 Figure. Uncropped gel shown in Figure 1c.
